# Implantation mycoses: A nationwide survey on diagnostic and treatment modalities in Nepal

**DOI:** 10.1371/journal.pntd.0014223

**Published:** 2026-04-20

**Authors:** Niraj Parajuli, Usha Kiran, Rushma Shrestha, Suwash Baral, Gokarna Dahal, Roushan Jahan, Sushil Paudel, Yogesh Poudyal, Rabindra Baskota, Prajwal Pudasaini, Hye Lynn Choi, Daniel Argaw Dagne, Barbara Milani

**Affiliations:** 1 Department of Dermatology and Venereology, National Academy of Medical Sciences, Bir Hospital, Kathmandu, Nepal; 2 Rare Skin Disease Nepal, Kathmandu, Nepal; 3 Consultant, Rare Skin Disease Nepal, Kathmandu, Nepal; 4 Anandaban Hospital, Lele, Lalitpur, Nepal; 5 NTD and Vector-Borne Section, Epidemiology and Disease Control Division, Ministry of Health and Population, Kathmandu, Nepal; 6 Department of Dermatology, Kanti Children’s Hospital, Kathmandu, Nepal; 7 Department of Dermatology, Civil Service Hospital, New Baneshwor, Kathmandu, Nepal; 8 Sarnath Skin Centre, Bhairahawa, Rupandehi, Nepal; 9 Department of Health Services, Ministry of Health and Population, Teku, Kathmandu, Nepal; 10 Department of Regulation and Prequalification [WHO/RPQ], World Health Organization, Geneva, Switzerland; 11 Department of Control of Neglected Tropical Diseases [WHO/NTD], World Health Organization, Geneva, Switzerland; 12 Consultant, Department of Control of Neglected Tropical Diseases [WHO/NTD], World Health Organization, Geneva, Switzerland; Albert Einstein College of Medicine, UNITED STATES OF AMERICA

## Abstract

Implantation mycoses (IM) are a group of fungal diseases which occur after a transcutaneous trauma. The World Health Organization has listed some of the IM, namely sporotrichosis, chromoblastomycosis and eumycetoma as one of the Skin Neglected Tropical Diseases targeted for control by 2030. There are no robust data on IM from Nepal since these diseases are not a part of routine disease surveillance. In this study, an online and in-person survey was conducted using a standard set of questionnaires among the registered dermatologists of Nepal. For this survey sporotrichosis, chromoblastomycosis and mycetoma were included. A total of 56 dermatologists responded to this survey. The result showed that sporotrichosis was the most diagnosed IM (46/56, 82.15%) whereas mycetoma was the least common (21/56, 37.5%). This study further explored the availability of the various diagnostics and treatment modalities among these IM in Nepal. It also showed a lack of uniformity in treatment modalities and in the availability of diagnostics as well as treatment options across the country. It showed that the diagnostics and treatment options were more in the capital as compared to rest of the country.

## Author summary

Implantation mycoses are a group of fungal diseases which are caused by cutaneous trauma. The data on these conditions are lacking in Nepal. We conducted a national level survey among registered dermatologists using a standard set of questionnaires to gather information on their experience in the diagnostic capacities and treatment practices. An in-person meeting including dermatologists across every major institute and all provinces was also conducted. A total of 56 dermatologists responded to this survey. The survey result showed sporotrichosis was the most common IM and highlighted a lack of uniformity in treatment as well as discrepancies on the availability of the diagnostics and treatment modalities across different regions.

## Introduction

Implantation mycoses (IM) are a heterogeneous group of fungal diseases that develop at the site of trauma [1]. The trauma can lead to the inoculation of the causative organism, either trivial or severe. As IM may also involve muscles, fascia, cartilages and bones, the previous nomenclature subcutaneous mycoses have been frequently replaced with the term IM in recent years. These mycoses are prevalent mainly among the low and low-middle income countries, and mostly in the rural regions of the tropics and subtropics. The most common IM are mycetoma, chromoblastomycosis and sporotrichosis. Most of these conditions are associated with significant morbidity and disability, which can be prevented in the majority of cases with early diagnosis and management [2,3].

Mycetoma is a chronic and progressively destructive form of IM which affects the subcutaneous tissue and can spread to affect the skin, deep tissues, and bone [4,5]. Mycetoma can either be actinomycetoma (caused by bacteria) or eumycetoma (caused by fungi). Although foot is the most affected region, it can affect any part of the body. More than 70 different fungi and bacteria have been found as the causative agents [2]. Mycetoma has numerous adverse medical and socioeconomic consequences for the patients, communities, and health services in the affected regions.

Chromoblastomycosis is a chronic infection caused by the traumatic inoculation of a group of dematiaceous fungi where *Fonsecaea pedrosoi, Cladophialophora carrionii and Phialophora verrucosa* are the most commonly implicated species [6,7]. Chromoblastomycosis mostly affects people in rural areas who work outdoors without proper footwear and protective clothing [8].

Sporotrichosis is an infection caused by dimorphic fungi of the genus *Sporothrix* [9]. It occurs in three main clinical forms: cutaneous, pulmonary, and disseminated. Sporotrichosis causes skin lesions that commonly present as chain of nodules but may also occur as single nodule or even ulcers [10].

The World Health Organization (WHO) has listed mycetoma, chromoblastomycosis and other deep mycoses as one of the skin- related neglected tropical diseases (Skin-NTDs). The WHO global NTD roadmap (2021–2030) aims to control mycetoma, chromoblastomycosis and other deep mycoses in the endemic countries by 2030. The roadmap targets to increase the number of countries in which mycetoma, chromoblastomycosis and sporotrichosis are included in national control programs and surveillance system from 3% countries as a baseline in 2020 to 50% by 2030. The roadmap has also identified few critical actions such as development of different rapid diagnostic tests and effective treatments; and establishment of surveillance for case detection and reporting; development of a standardized field manual for diagnosis and treatment and ensure proper training of health care workers and providing access to affordable diagnosis and treatment in order to achieve the set targets by 2030 [11]. It has also set global targets and milestones for these group of diseases or disease conditions which are prevalent in tropical and subtropical countries like Nepal.

In Nepal, the actual burden of IM is still unknown as neither a well-established routine surveillance system to capture this information nor any national control programs for IM exists. There is a need to understand the actual burden of IM as well as to know the current diagnostic and treatment modalities being practiced across the country. IM are difficult to diagnose and treat and cause significant morbidity and disability. These conditions are also associated with substantial social stigma and marked reduction in quality of life. Treatment of IM in Nepal is based on personal experience and case reports published outside the country. National standardized guidelines on diagnosis and management of implantation mycoses is not yet available in Nepal. It is expected that the results of this study will support the national health authorities on providing initial substantial evidence on the burden of IM in Nepal in order to develop national control plans of these skin NTDs.

## Methods

### Ethical approval

Ethical approval for this study was taken from Nepal Health Research Council, Kathmandu Nepal and the Ethical review committee of World Health Organization (WHO).

This study was conducted by Rare Skin Disease Nepal (RSDN), a non-governmental organization registered in Nepal in close collaboration with the Society of Dermatologists, Venereologists and Leprologists of Nepal (SODVELON) and Epidemiology and Disease Control Division (EDCD), Ministry of Health and Population Nepal (MoHP) with the general objective to collect information on the various diagnostics and treatment modalities of implantation mycoses in Nepal and the following specific objectives:

1. To find the burden of implantation mycoses in the surveyed areas in Nepal.
2. To find information on different diagnostic and treatment modalities in IM across different levels of health care settings and geographic region in Nepal.

### Study design

The study was conducted through a survey approach using a standard survey questionnaire. Survey questionnaire was adapted using the WHO survey questionnaire used for the global WHO online survey on diagnostic capacities and treatment practices for implantation mycoses [3]. The questionnaire was reviewed and adapted to the country context.

### Study participants

Registered dermatologists in Nepal willing to undertake the survey were included for this study. Since dermatologists are often the first specialists to see skin-related conditions, and because most patients in Nepal with skin diseases are referred directly to dermatologists rather than to other specialists, they were therefore selected as the primary respondents for this survey.

### Study period

This study was conducted between November 2023 and March 2024.

### Study methods

The survey questionnaires were completed through two approaches, onsite and online. Details of the study participants (registered dermatologists in Nepal) which included their names and email addresses, were requested and obtained from SODVELON email registry. The onsite survey participants completed the survey after signing the informed consent form in paper-based model while the online survey participants were provided survey questionnaire in google forms, the link of which was sent to their email addresses. The survey questionnaire included 16 questions which were in English language. Since the study participants were registered medical doctors (dermatologists) in Nepal whose main language of medical education and medical licensing examination is English, questionnaires were not translated into national language, Nepali. The onsite survey participants were invited to Kathmandu, the capital city of the country where they completed and submitted the survey questionnaire. The gathering of the onsite survey participants (dermatologists from major hospitals with high number of dermatological cases across all the seven provinces of the country) was also used as an opportunity to hold a meeting and discuss gaps and challenges for diagnosis and treatment of IM in Nepal. The onsite survey participants were provided with an informed consent form prior to starting the survey questionnaires. Online survey participants could proceed to the online survey questionnaire after providing consent to participate to take part in the survey electronically.

## Results

A total of 56 participants completed the survey among which 14 participants completed the survey through paper-based form and remaining 42 through online in google forms. The findings from the survey are provided below as per the 16 questions included in the survey questionnaire.

### Survey participants’ profile

**Fig 1:**
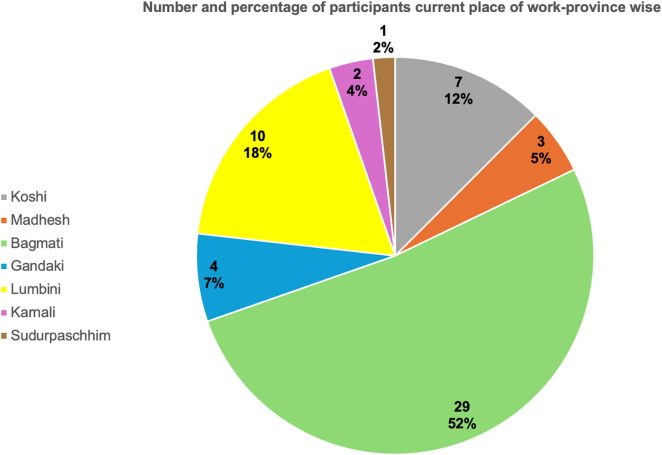
Province-wise distribution of participants by current place of work.

**Fig 2:**
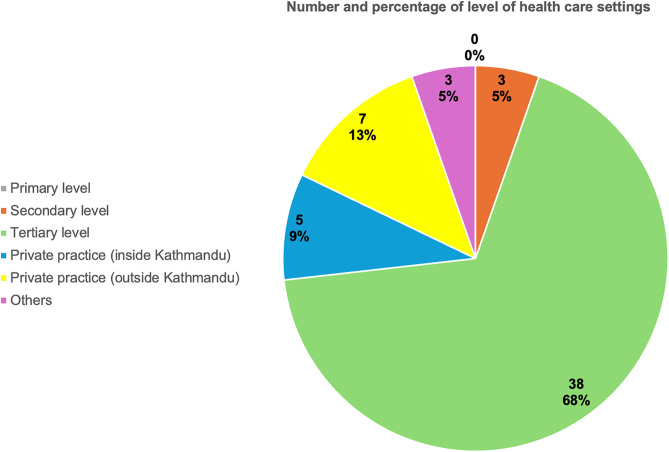
Distribution of participants by level of health care setting for their current place of work.

The participants’ current place of work covered all the seven provinces of the country. The maximum number of participants were from Bagmati province (51.8%) and least from far west, Sudurpaschhim province (1.8%). Fig 1 presents the number of survey participants according to the 7 provinces of Nepal. Out of the 56 participants who participated in the survey, maximum number of participants (~64%) were working at the tertiary level health institutions which included government institutions, semi- government, and private medical colleges (Fig 2). Around half of the participants were working in an academic center.

### Reported diagnosis of IM and available diagnostic techniques available

**Fig 3:**
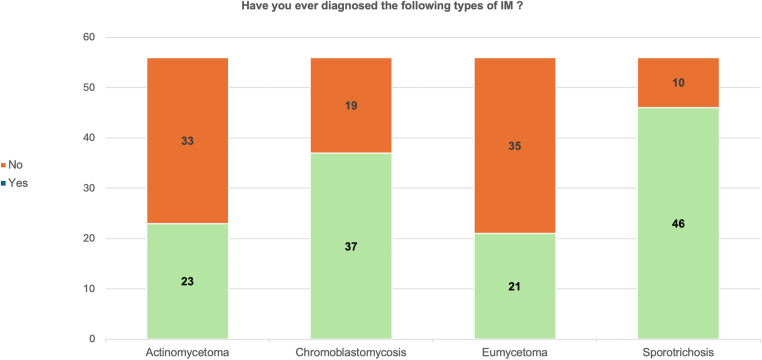
Number of participants who have diagnosed IM at their current workplace.

In response to the survey question “ Have you ever diagnosed one of the following IM in your current place of work?”, 82% (46/56) responded diagnosing sporotrichosis, 66% (37/56) responded diagnosing chromoblastomycosis, 41% (23/56) responded they have diagnosed actinomycetoma and 37.5% (21/56) mentioned on diagnosing eumycetoma as presented in Fig 3. Regarding the availability of various diagnostic techniques for IM, all participants used clinical features/visual inspection for suspecting the diagnosis. Histopathology was the most commonly available and utilized diagnostic modality which was utilized by more than 85% of the respondents. In contrast, neither serological nor molecular diagnostic methods (PCR) for any type of IM were available. Detail responses on the availability of various diagnostic modalities are presented in the Table 1.

**Table 1:** Availability of various diagnostic techniques for different IM types among the participant’s current workplace.

Diagnostic technique	Eumycetoma N (%)	Actinomycetoma N (%)	Chromoblastomycosis N (%)	Sporotrichosis N (%)
Direct microscopy	28 (50)	26 (46.4)	16 (28.5)	18 (32.1)
Culture	12 (21.4)	14 (25)	13 (23.2)	10 (17.8)
Serology	0 (0)	0 (0)	0 (0)	0 (0)
Molecular diagnosis (PCR)	0 (0)	0 (0)	0 (0)	0 (0)
Histopathology of skin biopsy	50 (89.2)	50 (89.2)	49 (87.5)	49 (87.5)
Dermoscopy: epiluminescence microscopy (ELM)	16 (28.5)	16 (28.5)	14 (25)	13 (23.2)
Others**	2 (3.5)	2 (3.5)	0 (0)	1 (1.8)

Others**: Radiological diagnosis including x-ray (to rule out bony involvement) and MRI.

### Reported burden of IM by the survey participants over the last year and the last three years

According to the participants’ responses, sporotrichosis (123 cases) was the most commonly diagnosed while actinomycetoma (33 cases) was the least diagnosed IM type over the previous one-year period (Table 2). Although all the participants have responded with the number of IM cases diagnosed in their workplace over the last one- year period, 37 of the 56 participants also mentioned that information on number of IM cases was not available in institutional records.

**Table 2:** Number of IM cases diagnosed in the participants center over last one year and last three years.

S.N.	IM type	Number of IM diagnosed (last one year)	Number of IM diagnosed (last three years)
1	Eumycetoma	40	64
2	Actinomycetoma	33	52
3	Chromoblastomycosis	78	112
4	Sporotrichosis	123	174
5	Information not available from institution records	37 participants responded that information not available from institution records	41 participants responded that information not available from institution records

Likewise, sporotrichosis (174 cases) was the most frequently diagnosed while actinomycetoma (52 cases) was the least frequently diagnosed IM type over the last three-year period. Although all the participants responded with the number of IM cases diagnosed in their workplace over the last three- year period, 41 of the 56 participants also mentioned that information on number of IM cases was not available from institutions records. The number of IM cases they provided for both one year and three years were from the participant’s own personal records of patients.

### Pharmacological and non-pharmacological treatment used for the diagnosed IM

Of the 56 participants, 21 responded that they had diagnosed eumycetoma in their current place of work. Oral itraconazole was the most preferred treatment used by all participants (100%) for the treatment of eumycetoma. None of the participants reported using oral posaconazole. The details on the medications used for the treatment of eumycetoma in the participants’ current workplace are presented in Table 3. A total 9 out of 21 participants responded that they do not apply any non-pharmacological intervention for the treatment of eumycetoma and 8 out of 21 responded they perform surgery. Details on other non-pharmacological interventions they had applied for the treatment of eumycetoma are presented in Table 4.

**Table 3:** Medications used for the treatment of eumycetoma by the participant’s at their workplace.

S.N.	Medication	Number	Percentage %
1	Itraconazole (oral)	21/21	100
2	Posaconazole (oral)	0/21	0
3	Voriconazole (oral)	2/21	9.5
4	Ketoconazole (oral)	1/21	4.7
5	Terbinafine (oral)	7/21	33.3
6	Amphotericin B (injectable)	2/21	9.5
7	Ayurvedic medicine. Please indicate	0/21	0
8	Natural medicine: Please indicate	0/21	0
9	Not applicable (If you have not treated this disease)	35/56	62.5

**Table 4:** Different non-pharmacological interventions applied for the treatment of eumycetoma by the participants.

S.N.	Non-pharmacological intervention	Number	(%)
1	Surgery	8/21	38.1
2	Heat therapy	2/21	9.5
3	Cryotherapy/cryosurgery	5/21	23.8
4	Laser phototherapy	1/21	4.7
5	Photodynamic therapy (PDT)	0/21	0
6	None	9/21	42.8
7	Others: Please comment	0/21	0
8	Not applicable (If you have not treated this disease)	35/56	62.5

A total of 23 participants responded that they had diagnosed actinomycetoma in their current workplace. Trimethoprim-sulfamethoxazole (oral) (91.3%) was the mostly used medicine for the treatment of actinomycetoma followed by amoxicillin/ clavulanic acid (oral) (65.2%). Fosfomycin and moxifloxacin were not used at all. Neither ayurvedic medicine nor natural medicine were used for its treatment by the participants. One of the participants responded that oral doxycycline was used for the treatment of actinomycetoma in the participants current workplace. Details on the use of pharmacological interventions for the treatment of actinomycetoma is presented in Table 5.

**Table 5:** Medicines used for the treatment of actinomycetoma by the participants in their current workplace.

S.N.	Medication	Number	Percentage %
1	Trimethoprim-sulfamethoxazole (oral)	21/23	91.3
2	Amikacin (IV)	6/23	26.1
3	Amoxicillin/ Clavulanic acid (oral)	15/23	65.2
4	Rifampicin (oral)	9/23	39.1
5	Dapsone (oral)	7/23	30.4
6	Moxifloxacin (oral)	0/23	0
7	Carbapenems (IV)	2/23	8.7
8	Fosfomycin (oral)	0/23	0
9	Ayurvedic medicine. Please indicate	0/23	0
10	Natural medicine: Please indicate	0/23	0
11	Others: Please comment	1/23	4.3
12	Not applicable (If you have not treated this disease)	33/56	58.9

Others: oral doxycycline.

A total of 37 participants responded that they had diagnosed chromoblastomycosis in their current workplace. Itraconazole (oral) (100%) was the most commonly prescribed medicine for the treatment of chromoblastomycosis followed by Terbinafine (oral) (32.4%). Details on other medications are presented in Table 6. Majority of the participants (37.8%) responded that they do not use any non-pharmacological intervention for the treatment of chromoblastomycosis. Around 30% of the participants used heat therapy and cryotherapy/cryosurgery. Details on use of other non-pharmacological interventions are presented in Table 7. Refractory cases were also noted by some participants. These are defined as disease not improving with long duration of systemic therapy [7]. Of the 37 participants who responded that they had diagnosed chromoblastomycosis in their current workplace, 13/37 (35.1%) participants responded that they have recorded refractory cases of chromoblastomycosis in their work settings (Table 8).

**Table 6:** Medicines used for the treatment of chromoblastomycosis in the participant’s current place of work.

S.N.	Medication	Number	Percentage %
1	Itraconazole (oral)	37/37	100
2	Posaconazole (oral)	0/37	0
3	Voriconazole (oral)	0/37	0
4	Flucytosine (oral)	0/37	0
5	Imiquimod (topical)	0/37	0
6	Ayurvedic medicine. Please indicate	0/37	0
7	Natural medicine: Please indicate	0/37	0
8	Other: Please comment	3/37	8.1
9	Not applicable (If you have not treated this disease)	19/56	33.9

Others: Oral supersaturated solution of potassium iodide

**Table 7:** Non-pharmacological interventions applied for the treatment of chromoblastomycosis in the participants’ current place of work.

S.N.	Non- pharmacological interventions	Number	Percentage %
1	heat therapy	11/37	29.7
2	Cryotherapy/cryosurgery	11/37	29.7
3	Laser phototherapy	4/37	10.8
4	Photodynamic therapy (PDT)	0/37	0
5	Other: Please comment*	2/37	5.4
6	None	14/37	37.8
7	Not applicable (If you have not treated this disease)	19/56	33.9

*Others: surgical excision

**Table 8:** Number of refractory cases of chromoblastomycosis among the participant’s current workplace.

S.N.	Response	Number	Percentage %
1	Yes	13/37	35.1
2	No	24/37	64.8
3	Not applicable (If you have not treated this disease)	19/56	33.9
	**Total response**	**56**	**100**

Forty-six of the 56 participants who took the survey had responded that they have diagnosed sporotrichosis in their current workplace. Oral itraconazole was the most prescribed medicine (45/46) followed by potassium iodide (oral) (23/46) for the treatment of cutaneous sporotrichosis as responded by the participants. The details on use of other medicines are presented in Table 9. Refractory cases are defined as disease not improving even after 4 months of systemic therapy [12]. Majority of the participants (86.9%) responded that they have no refractory cases of sporotrichosis while 6/46 (13%) responded that there are refractory cases of sporotrichosis in their current workplace (Table 10).

**Table 9:** Medicines used for the treatment of cutaneous sporotrichosis in the participants’ current place of work.

S.N.	Medication	Number	Percentage %
1	Itraconazole (oral)	45/46	97.8
2	Terbinafine (oral)	12/46	26
3	Potassium iodide (oral)	23/46	50
4	Ayurvedic medicine. Please indicate	0/46	0
5	Natural medicine: Please indicate	0/46	0
6	Others, please comment	1/46	2.1
7	Not applicable (If you have not treated this disease)	10/56	17.8

Others: cryotherapy

**Table 10:** Number of refractory cases of cutaneous sporotrichosis at the respondent’s workplace.

S.N.	Response	Number	Percentage %
1	Yes	6/46	13.04
2	No	40/46	86.9
3	Not applicable (If you have not treated this disease)	10/56	17.8
	**Total response**	**56**	**100**

### Medicines that are readily available for use in the participants area

All the participants (56) responded that itraconazole (oral) was readily available for use in their area which meant the ease of access to these medications. More than 85% responded that oral terbinafine was also readily or easily available, whereas oral posaconazole and topical imiquimod were readily available only in around 7% and 5% respectively in the participants area. Medicines such as oral trimethoprim-sulfamethoxazole and oral amoxicillin/clavulanic acid were readily available in around 85% of the participants work area. Fosfomycin (oral) and flucytosine (oral) were not available in any participant’s work settings. The details on all medicines in the order of readily available to least available are presented in Table 11.

**Table 11:** Medicines which are easily available for use in the participants area.

S.N.	Medicine	Readily available for use Yes (number)	Percentage %
1	Itraconazole (oral)	56	100
2	Trimethoprim-sulfamethoxazole (oral)	49	87.5
3	Terbinafine (oral)	48	85.7
4	Amoxicillin/ Clavulanic acid (oral)	48	85.7
5	Ketoconazole (oral)	39	69.6
6	Amikacin (IV)	37	66.1
7	Voriconazole (oral)	30	53.5
8	Dapsone (oral)	29	51.8
9	Rifampicin (oral)	28	50
10	Potassium iodide (oral)	20	35.7
11	Moxifloxacin (oral)	17	30.3
12	Carbapenems (IV)	13	23.2
13	Amphotericin B (injectable)	13	23.2
14	Posaconazole (oral)	4	7.15
15	Imiquimod (topical)	3	5.36
16	Fosfomycin (oral)	0	0
17	Flucytosine (oral)	0	0

### Gaps and challenges outlined by the survey participants

During the onsite meeting with the selected dermatologists from across the country, gaps and challenges on three aspects related to implantation mycosis- surveillance, diagnostic techniques, and treatment were discussed. Few major gaps and challenges highlighted during the meeting is presented in the Table 12 below:

**Table 12:** Gaps/challenges and way forward identified for Implantation Mycosis.

IM areas	Gaps/challenges
**Surveillance**	The National Health Information System does not capture IM data from both government as well as private health institutions.
**Diagnosis**	Lack of laboratory facilities with availability of special tests such as molecular test (PCR) and culture Lack of laboratory facilities for species identification of fungus Limited trained human resources Delay in diagnosis due to lack of proper interdepartmental referral system
**Treatment**	Limited and not uniform availability of many efficacious IM medicines. Lack of standardized national treatment guideline. High economic burden to patients due to longer treatment duration and high costs of the IM medications.

## Discussion

This study used a survey questionnaire to assess the various diagnostic and treatment modalities for different types of IM in Nepal as well as to estimate their burden. The diagnostic techniques and treatment modalities for different IM were well captured in this survey because the respondents represented centers across the country where dermatological diseases are being diagnosed and treated. However, as IM cases are not systematically recorded in the hospital information systems, the true burden of these diseases are being underestimated. This first national survey on IM in Nepal offers valuable insights into the disease burden and highlights the key challenges in their management.

Most dermatologists who responded to the survey were from Bagmati province, where the capital city, Kathmandu, is located. This likely reflects the concentration of dermatologists in the capital which has a larger population and more hospitals than other provinces. Additionally, most of the participants were working at a tertiary-level health facilities, likely due to the absence of specialist positions, such as dermatologists in secondary level institutions (district hospitals). Although 35 participants (62.5%) were working in academic centers, publications on IM from Nepal still remains limited.

Sporotrichosis was the most commonly diagnosed IM in this survey, consistent with findings from the global survey conducted by WHO [13]. However, 17.8% of respondents had never diagnosed sporotrichosis in their clinical practice, despite it being one of the commonest IM globally [14]. Publications on IM from Nepal are limited to few case reports and case series [15–20]. The substantial number of cases of IM reported by the participants through this survey highlights the need for a national data recording and reporting system for IM. The reported figures were based on participants’ personal treatment records rather than hospital data. This underscores a significant gap in published data on IM in Nepal, as existing literature on IM and skin NTDs largely lacks accurate national data [9,21–23]. While the global burden of chromoblastomycosis includes some reported cases from Nepal, data on sporotrichosis and mycetoma remain largely absent [23].

There was a significant gap in the availability of diagnostic modalities for IM across the country. Clinical examination was used as the primary diagnostic method for suspecting and diagnosing all IM, followed by histopathological examination (87.2-89.5%). These findings are consistent with the WHO global survey, where the same diagnostic approaches were most frequently reported.[3] Although direct microscopic examination of grains is a cost-effective and reliable method for diagnosing mycetoma, its use was limited. Similarly, direct microscopy for chromoblastomycosis, despite being inexpensive, remained underutilized in Nepal. The availability of bacterial and fungal culture facilities was also minimal and advanced molecular diagnostic methods like PCR and serological assays were not available in any of the respondent’s workplace. Approximately one-fourth of the participants reported using dermoscopy for diagnostic purposes. A small number also mentioned on the use of radiological modalities including x-rays and magnetic resonance imaging highlighting their importance for accurate diagnosis and assessment of disease extent in various IM.

Oral itraconazole was the most commonly prescribed antifungal, used by all respondents for the treatment of eumycetoma and chromoblastomycosis. Nearly all respondents (97.8%) also reported using of oral itraconazole for sporotrichosis in line with the global trends^3^. Terbinafine was used by approximately one-third of the respondents for eumycetoma and chromoblastomycosis and by one-fourth for sporotrichosis. Among the newer generation azoles, posaconazole was not used, while voriconazole use was reported by only two participants for treatment of eumycetoma. This discrepancy of the anti-fungal preference for the treatment of IM is likely influenced by drug availability. Oral itraconazole is widely accessible, whereas newer antifungal agents are typically limited to larger urban centers. Additionally, the higher cost of the newer antifungals represents a significant barrier to their use as compared to more established therapies.

For actinomycetoma, treatment was primarily with oral trimethoprim-sulfamethoxazole (91.3%) and oral amoxicillin/clavulanic acid (65.2%), with limited parenteral use of IV amikacin (26.08%) and IV carbapenem (8.6%). No respondents reported using ayurvedic or other natural medicines, likely because these are not prescribed by clinical dermatologists. Non-pharmacological and adjunctive therapies were also employed, with surgery being the most commonly used.

For chromoblastomycosis, treatment modalities included cryosurgery (29.7%), heat therapy (29.7%) and laser therapy (10.8%), while a small number of respondents reported surgical excision and supersaturated solution of potassium iodide (SSKI) (5.36%). Refractory cases of chromoblastomycosis were also reported, raising concerns that these cases require further evaluation including assessment of drug resistance and careful management with combination therapies.

For eumycetoma, surgery was the most commonly used non-pharmacological intervention (32.15%) followed by cryosurgery (16%) and heat therapy (8.9%), although 29% of respondents reported not using any of these modalities.

In cutaneous sporotrichosis, oral itraconazole was the preferred treatment (87.5%) followed by SSKI (42.85%) and oral terbinafine (28.4%). Despite being a preferred option, SSKI’s limited availability restricted its use even in Nepal [14]. Six participants (11%) reported managing refractory sporotrichosis, a concern given the lack of fungal culture and sensitivity facilities nationwide. Oral itraconazole was widely available, whereas other preferred medicines such as oral terbinafine and potassium iodide were limited in availability across the country. Oral fosfomycin and flucytosine were unavailable in all surveyed workplaces. Newer modalities like topical imiquimod and oral posaconazole were accessible to only few participants, likely reflecting regulatory barriers and high out-of-pocket costs. For actinomycetoma, trimethoprim-sulfamethoxazole and amoxicillin-clavulanic acid were readily available nationwide. While accessibility is beneficial, it also raises concerns about the potential antimicrobial resistance as even the anti-microbials can be purchased as over the counter medicine in the country.

This survey provides a comprehensive overview of IM from Nepal, highlighting key gaps and challenges. A major finding was the absence of a surveillance system to capture the burden of IM within the existing national health information system. Diagnostics capacity is limited, with histopathological examination not uniformly available across the country. There are no centers performing specialized fungal tests and a shortage of trained personnel performing those. Access to recommended medications is inconsistent, with newer antifungals available only at select centers. Consequently, most cases were managed individually, as standardized national treatment guidelines are lacking.

The in-person discussions emphasized potential strategies to address these gaps and challenges. Establishing an integrated surveillance system within the national health information platform is critical requiring collaboration between the Ministry of Health and population, healthcare providers and stakeholders. Data from such a system can guide policy, plan national strategies and support standardized disease management. Proper orientation and training of dermatologists and other health care workers, along with specialized fungal laboratories, is essential to improve diagnostics and mitigate antimicrobial resistance [24,25]. Only through a collective effort involving policy makers, health care providers and relevant stakeholder, we will be able to achieve the targets set for the control and elimination of various skin NTDs including IM [26].

This survey serves as a foundational assessment, identifying priority actions to address the largely unrecognized burden of IM. We hope that this work will serve as a foundation for further research on the IM and other skin-NTDs. Based on the findings, stakeholders working across neglected tropical diseases should join hands to fill the existing gaps and advance the control of implantation mycoses in Nepa**l.** Limitations include reliance on the participant-reported data, absence of patient demographic details and the inability to determine the exact causes of implantation due to the retrospective design. These findings underscore the need for further research and coordinated efforts to advance the control of IM and other skin-NTDs in Nepal.
